# The Genotype and Phenotype Features in a Large Chinese MFN2 Mutation Cohort

**DOI:** 10.3389/fneur.2021.757518

**Published:** 2021-10-13

**Authors:** Yan Ma, Aping Sun, Yingshuang Zhang, Dongsheng Fan, Xiaoxuan Liu

**Affiliations:** ^1^Department of Neurology, Peking University Third Hospital, Beijing, China; ^2^Beijing Municipal Key Laboratory of Biomarker and Translational Research in Neurodegenerative Diseases, Beijing, China; ^3^Key Laboratory for Neuroscience, Ministry of Education/National Health Commission, Peking University, Beijing, China

**Keywords:** Charcot–Marie–Tooth disease type 2A, *mitofusin2*, phenotype, genotype, *de novo* variants

## Abstract

**Introduction:** Charcot–Marie–Tooth disease type 2A (CMT2A) is a group of clinically and genetically heterogeneous disorders, which is mostly caused by mutations of the *mitofusin2* (MFN2) gene. As the genotype–phenotype characteristics of CMT2A were still incompletely understood, we further explored the spectrum of CMT2A variants in China and demonstrated their phenotypic diversities.

**Methods:** A total of 402 index patients/families with CMT throughout Mainland China were enrolled in this study. Among them, we analyzed 20 unrelated index cases with CMT2A by Sanger sequencing, next-generation sequencing, or whole-exome sequencing. Detailed clinical and genetic features of CMT2A patients were collected and analyzed. Of note, *de novo* mutations were not rare in MFN2 gene; we compared the clinical features of patients from the *de novo* group with those from the non-*de novo* group.

**Results:** We identified 20 MFN2 variants, occupying 5.0% of CMT. Most patients presented with early onset and moderate phenotype with abnormal gait and foot drop as the main complaints at onset. Pyramidal signs accounts for 31.6% (6/19) in all patients, which is not uncommon. Four novel variants (p.Tyr752^*^, c.475-2A>G, p.Val99Met, and p.Arg275_Gln276insArg) were identified in the cohort. Besides, *de novo* variants occupied 35.0% (7/20) in our study with a much earlier age at onset compared with those in the non-*de novo* group (*p* = 0.021).

**Conclusion:** Chinese CMT2A is a predominant typical pure CMT2A, with early onset and mild to moderate phenotype. Given the high frequency of *de novo* MFN2 mutations, genetic study should be considered for patients with early onset and severe idiopathic axonal neuropathy.

## Introduction

Charcot–Marie–Tooth disease (CMT) is one of the most common inherited peripheral neuropathies with a prevalence of 1/2,500. Generally, CMT is classified into a demyelinating type, an axonal type, and an intermediated type according to the motor nerve conduction velocity (MCV) of the median nerve. Charcot–Marie–Tooth disease type 2A (CMT2A) is the most frequent subtype of axonal CMT. In literature, the contribution of CMT2A to Charcot–Marie–Tooth disease type 2 (CMT2) are strikingly varied, ranging from 8 to 30% (1–7) among different ethnic origins. As a group of heterogeneous disorders, the clinical manifestations of CMT2A are varied from severe, early-onset to mild, and late-onset axonal neuropathy. The classic clinical features of CMT2A (pure CMT2A) comprised of progressive distal muscle weakness, atrophy, mild sensory loss, depressed tendon reflexes, and deformity. To date, more than a few CMT2A patients presented complex phenotypes (complex CMT2A), involving pyramidal signs, deafness, optic atrophy, tremor, ataxia, vocal cord involvement, and white matter lesions on MRI, which made the diagnosis more complex (8). In previous studies, the phenotypic features of CMT2A remain less clear. CMT2A ranged from a mild to severe form with two peaks of onset age according to different patients series (1, 9, 10).

Mitochondria play an important role in aerobic metabolism especially in neurons. *Mitofusin2* (MFN2) is a GTPase dynamin-like mitochondrial membrane proteins, which plays an essential role in mitochondrial dynamics and axonal traffic (11). Since MFN2 gene has been recognized to be a major cause of CMT2A, more than 100 disease-associated mutations and many variants of unknown significance of MFN2 have been identified (12), which broaden the phenotypic spectrum of MFN2-related neuropathy. Besides, interfamilial or intrafamilial variability is also evidence in some special mutation sites, which made the diagnosis more challenging. Therefore, a better genotype–phenotype correlation study may benefit the gene screening for clinically suspicious patients. In addition, although most pathogenic variants in MFN2 are inherited in a dominant pattern, *de novo* cases of CMT2A are not rare and have been reported in a few cases. However, the phenotypic characteristics of *de novo* MFN2 variants remain incompletely understood.

Thus, to further explore the spectrum of MFN2 variants, we sequenced the MFN2 gene in a large population of CMT index cases in Mainland China. Besides, we also attempted to summarize the features of *de novo* MFN2 variants, in order to give some clues for clinicians in directing future diagnosis.

## Materials and Methods

### Subjects

A total of 402 index patients/families with CMT throughout Mainland China were consecutively recruited at Peking University Third Hospital from 2007 to 2020. Among them, 39 patients from 20 unrelated families were diagnosed with CMT2A out of 159 axonal CMT patients. Of the 20 families, 11 were compatible with an autosomal dominant mode of inheritance with a positive family history, while nine showed isolated pattern. CMT2 was diagnosed according to the well-established clinical criteria when the median nerve MCV was >42 m/s and accompanied with clinical features (13). An experienced neurologist evaluated clinical symptoms and physical examinations of all patients and mutation carriers with MFN2 gene variants. The disease burden was assessed by the CMT Neuropathy Scores (CMTNS). On the basis of the CMTNS, patients can be classified as having mild, moderate, or severe disease (CMTNS of ≤ 10, 11–20, or >20, respectively). As the CMTNS may be influenced by the course of the disease, we use the CMTNS Gradient (CMTNS-G), measured as the ratio of the CMTNS at examination to the duration of disease, to evaluate the disease course. The institutional ethics committee of Peking University Third Hospital approved this study. All patients and family members provided written informed consent to participate in this study.

### Gene Sequencing and Variant Interpretation

Genomic DNA was collected and extracted from peripheral blood leukocytes using DNA Isolation Kit (Bioteke, AU1802). From 2007 to 2013, MFN2 (NM_014874) mutations were screened by direct Sanger sequencing for suspected axonal CMT patients. After 2013, we used either a next-generation gene panel covering 404 peripheral neuropathy related genes or a whole-exome sequencing (WES) on the index patients/independent families. The samples were sequenced on the HiSeq X10 and NEXTSEQ 500 (Illumina, San Diego, CA, USA) to discover causal genes.

Identified MFN2 gene variants in next-generation sequencing (NGS) were further validated by Sanger sequencing. We confirmed all previously reported pathogenic mutations with reference to the Human Gene Mutation Database (HGMD) (http://www.hgmd.cf.ac.uk/ac/index.php). Novel variants were checked with the SNP database (dbSNP, http://www.ncbi.nlm.nih.gov/projects/SNP), the 1,000 Genomes Project (http://www.internationalgenome.org/) and Exome Aggregation Consortium (ExAC) (http://exac.broadinstitute.org). The potential impact of missense mutations on the structure and function of the encoded proteins were evaluated by MutationTaster (http://www.mutationtaster.org), PolyPhen-2 (http://genetics.bwh.harvard.edu/pph2) and SIFT (http://sift.jcvi.org/). Moreover, novel variants were interpreted and classified according to the American College of Medical Genetics and Genomics/Association for Molecular Pathology (ACMG/AMP) standards and guidelines (14).

### Statistical Analysis

All statistical analyses were performed using GraphPad Prism 7.0 (GraphPad Software, Inc., CA, USA) and SPSS V.23.0 software (IBM Corp., Armonk, USA). Descriptive statistics such as age at onset (AAO), electromyography (EMG) parameters, CMTNS, and CMTNS-G were expressed as mean (SE) or median (range). Moreover, comparison of continuous variables that were not normally distributed was analyzed using non-parametric tests, while a standard chi-square test or Fisher's exact test was used to analyze dichotomous variables, such as gender. A two-tailed *p* < 0.05 was considered statistically significant.

## Results

### Overview of Clinical Features of the Patient Cohort

Among all 402 CMT patients, we analyzed 159 unrelated CMT2 cases and identified 16 MFN2 gene variants in 20 isolated families (male:female = 1.5:1). Therefore, CMT2A occupied 12.6% of axonal CMT and 5.0% of all CMT. The median AAO of all probands was 8 years old (range from 2 to 54 years old), with 78.9% (15/19) cases developed clinical symptoms earlier before their second decade of life. Except for one patient diagnosed with distal hereditary motor neuropathy (dHMN), the others presented as typical CMT2 subtype. In general, clinical features in our CMT2A cohort contained slowly progressive walking difficulties, falls, weakness, and atrophy of the distal lower limbs with mild sensory involved. According to the clinical characteristics, we classified CMT2A into two clinical subtypes. Pure CMT2A refers to classic axonal CMT2 while complex CMT2A containing other symptoms such as optic atrophy, hearing loss, and pyramidal signs. In our CMT2A cohort, 68.4% (13/19) presented as pure subtype, while 31.6% (6/19) are complex CMT2A (decreased vision, hearing loss, pyramidal signs, and decreased memory). Abnormal gait and foot drop are main complaints as the symptoms at onset. Upper limbs involvement was seen in 38.9% (7/18) patients, in which six patients manifested as early onset subtype while one patient (F19) with splicing variant (c.475-2A>G) showed a late onset pattern. Sensory loss was seen in 66.7% (8/12) patients with lower limbs predominant. Among all patients, three underwent orthopedic surgery. In physical examinations, the flexor muscles of the legs were even more disabled than the extensor muscles. Pyramidal signs accounts for 31.6% (6/19) in all patients, which is not uncommon. By contrast, decreased vision and hearing loss were only seen in one patient, respectively, which seems rare in our study. Besides, a female patient carrying the p.Gln367Pro mutation has a complaint of memory loss at the age of 37. On the base of CMTNS (median = 12, range from 5 to 19), patients can be classified as mild (38.5%, 5/13) and moderate (61.5%, 8/13). In order to avoid the influence of disease duration, we used CMTNS-G (median = 1.25, range from 0.28 to 4) to evaluate the disease course. The detailed clinical features are summarized in Table 1.

**Table 1:** Clinical information of 20 identified *mitofusion2* (MFN2) pedigrees in our cohort.

**Proband**	**DNA changes**	**Amino acid** **changes**	**Gender/** **age (years)**	**AAO** **(years)**	**Disease** **duration** **(years)**	**Muscle** **atrophy** **(UL/LL)**	**Muscle** **weakness** **(UL/LL)**	**Pyramidal** **Sign**	**Sensory** **loss** **(UL/LL)**	**Other** **Signs**	**CMTNS/** **CMTNS-G**
F1	c.280C>T	p.Arg94Trp	M/6	4	2	N/Y	N/Y	Y	N/Y	Decreased vision	8/4
F2	c.919A>G	p.Lys307Glu	M/27	13	14	N/Y	N/Y	N	N/N	/	12/0.86
F3	c.775C>T	p.Arg259Cys	M/14	10	4	N/Y	N/Y	Y	N/N	dHMN	5/1.25
F4	c.475-1G>A	Splicing	M/27	20	7	N/Y	N/Y	N	Y/Y	/	14/2
F5	c.1126A>G	p.Met376Val	M/39	3	36	Y/Y	Y/Y	N	N/N	/	10/0.28
F6	c.775C>T	p.Arg259Cys	M/27	18	9	N/Y	N/Y	N	Y/Y	/	11/1.22
F7	c.295G>A	p.Val99Met	F/26	25	4	N/Y	N/Y	N	N/Y	/	10/2.5
F8	c.1100A>C	p.Gln367Pro	M/7	4	3	N/Y	N/Y	N	–	/	–
F9	c.280C>T	p.Arg94Trp	M/41	3	38	N/Y	N/Y	N	–	/	–
F10	c.828G>C	p.GLn276His	F/37	9	28	Y/Y	Y/Y	N	–	/	14/0.5
F11	c.746C>T	p.Ser249Phe	F/32	4	28	Y/Y	Y/Y	N	–	/	
F12	c.751C>T	p.Pro251Ser	M/2	2	–	N/Y	N/Y	Y	–	/	–
F13	c.280C>T	p.Arg94Trp	F/20	–	–	–	–	–	–	/	–
F14	c.1091G>A	p.Arg364Gln	F/-	25	–	–	–	N	–	/	–
F15	c.730G>C	p.Val244Leu	F/5	3	2	Y/Y	Y/Y	N	–	/	–
F16	c.826_827insGGC	p.Arg275_Gln276insArg	M/29	16	13	N/Y	N/Y	N	N/Y	/	17/1.31
F17	c.2256C>G	p.Tyr752*	M/19	5	14	Y/Y	Y/Y	Y	Y/Y	Hearing loss	19/1.36
F18	c.281G>A	Arg94Gln	F/30	8	22	N/Y	N/Y	N	Y/Y	/	12/0.43
F19	c.475-2A>G	Splicing	M/57	54	3	Y/Y	Y/Y	Y	N/N	/	5/1.67
F20	c.1100A>C	p.Gln367Pro	F/37	6	31	Y/Y	Y/Y	Y	N/Y	Decreased memory	18/0.58

*AAO, age at onset; UL, upper limbs; LL, lower limbs; CMTNS, Charcot–Marie–Tooth disease neuropathy scores; CMTNS-G, Charcot–Marie–Tooth disease neuropathy scores gradient*.

The electrophysiological findings of 16 patients are summarized in Table 2. The MCV of the median nerve was 58.09 ± 1.79 m/s, and the compound muscle action potential (CMAP) was 6.31 ± 0.88 mV. The MCV of the ulnar nerve was 55.42 ± 1.81 m/s and the CMAP was 4.56 ± 0.73 mV. Besides, three patients who had no clinical sensory deficit showed decreased sensory nerve action potential (SNAP) amplitude in our study. In addition, the lower limbs were severely affected, with more lower and absence of CMAP and SNAP amplitude.

**Table 2:** The electrophysiological findings of MFN2 pedigrees in our cohort.

**Proband**	**Gender/** **age (years)**	**Median nerve**		**Ulnar nerve**		**Tibial nerve**		**Median nerve**	**Ulnar nerve**	**Sural nerve**
		**CMAP** **(mV)**	**MCV** **(m/s)**	**CMAP** **(mV)**	**MCV** **(m/s)**	**CMAP** **(mV)**	**MCV** **(m/s)**	**SNAP (uV)/** **SCV (m/s)**	**SNAP (uV)/** **SCV (m/s)**	**SNAP (uV)/** **SCV (m/s)**
F1	M/6	5.0	45.8	/	/	0.06↓	29.6	4.5↓/41.1	/	A
F2	M/27	7.5	52.3	/	/	0.4↓	37.0	5.5/51.5	/	A
F4	M/27	10.1	62.1	8.0	65.9	A	A	4.7↓/53.4	A	A
F5	M/39	8.6	/	4.1	/	A	A	3.5↓/45.5	5.0/53.0	0.8↓/49.0
F6	M/27	9.4	57.0	6.5	55.6	0.025↓	37.1	2.6↓/48.1	0.3↓/48.6	A
F7	F/26	8.1	73.8	7.1	60.2	1.02↓	44.9	9.9/57.9	7.8/67.4	2.6↓/63.4
F8	M/7	6.6	57.7	7.1	56.1	0.3↓	17.5	/	/	1.4↓/35.3↓
F9	M/41	3.7↓	51.2	2.5↓	53.9	0.4↓	37.9	4.3↓/45.7	5.8/39.7	A
F10	F/37	1.2↓	56.8	2.8↓	51.2	A	A	6.2/53.8	1.7↓/51.9	A
F12	M/2	/	/	/	/	A	A	/	/	A
F14	F/25	12.8	56.0	/	/	0.3↓	33	3.0↓/51.0	/	A
F16	M/29	5.4	56.1	1.6↓	49.0	A	A	1.0↓/48.3	2.5↓/48.1	A
F17	M/19	1.45↓	66.7	1.67↓	43.5	A	A	A	A	A
F18	F/30	8.1	60.2	5.6	58.5	0.13↓	33.4	2.8↓/46.1	1.85↓/41.6	2.1↓/41.8
F19	M/57	5.1	58.3	6.6	58.7	0.9	38.9	8.7/62.5	7.4/62.5	10.0/50.0
F20	F/37	1.61↓	59.2	1.15↓	57.0	A	A	A	A	A

*CMAP, compound muscle action potential; SNAP, sensory nerve action potential; MCV, motor nerve conduction velocity; SCV, sensory nerve conduction velocity; A, absent. ↓The values were decreased than normal*.

### Genetic Analysis

**Figure 1:**
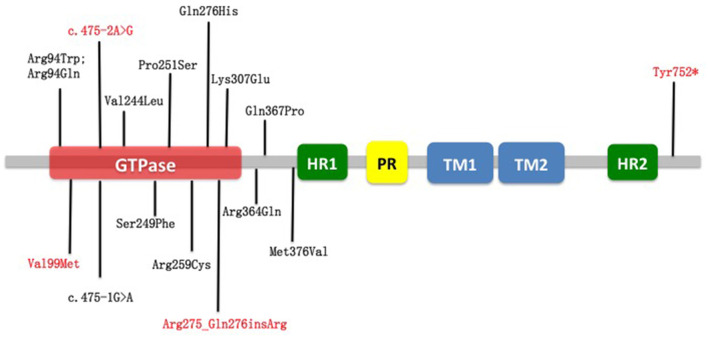
Schematic representation of MFN2 and localization of variants reported in this study. In red are novel variants on MFN2 detected in this study. In black are variants already reported. Nearly all variants were around the large GTPase domain near the N-terminus. GTPase, guanosine triphosphatase; HR1, heptad repeat 1; HR2, heptad repeat 2; PR, proline-rich; TM1, transmembrane domain 1; TM2, transmembrane domain 2.

Among all 16 identified MFN2 variants in our cohort, 12 are known pathogenic mutations, while four variants are novel (Table 3). A total of 12 missense, one frameshift, one nonsense, and two splicing variants were found (Table 1). From structural insights, 12 mutations were around the large guanosine triphosphatase (GTPase) domain near the *N*-terminus (Arg94Trp, Arg94Gln, Val99Met, c.475-2A>G, Arg275_Q276insR, Lys307Glu); one mutation was located adjacent to the C-terminus heptad repeat 2 (HR2) domain (Tyr752^*^), and three mutations were in the downstream region before the heptad repeat 1 (HR1) domain (Arg364Gln, Gln367Pro, Met376Val) (Figure 1). Of note, patients with p.Arg94Trp, p.Arg259Cys, p.Pro251Ser, p.Tyr752^*^, p.Gln367Pro, and c.475-2A>G showed a pyramidal phenotype, which is not uncommon in our cohort. The amino acid positions p.Arg94, p.Arg259, and p.Gln367 are the three commonest residues for the occurrence of missense variants, among which loci 94 in exon 4 was the hot spot mutation loci, accounting for 20% (4/20) of mutation-positive cases. Variants at specific amino acid positions that are usually associated with an early onset subtype.

**Table 3:** Molecular analysis and predicted pathogenicity of novel variants in our cohort.

**Exon**	**DNA changes**	**Amino acid changes**	**Database**			***In silico*** **analysis**			***De novo***	**GERP**	**ACMG**	**Phenotype**
			**dbSNP**	**ExAc**	**1000G**	**Poyphen-2**	**SIFT-2**	**Mutation Taster**				
4	c.295G>A	p.Val99Met	–	0	0	Probably damaging	Damaging	Disease causing	No	5.5	LP	Pure CMT2A
19	c.2256C>G	p.Tyr752*	rs863224968	0	0	–	–	Disease causing	Yes	5.39	P	Complex CMT2A
9	c.826_827insGGC	p.Arg275_Gln276insArg	–	0	0	–	–	Disease causing	No	–	LP	Pure CMT2A
intro5	c.475-2A>G	Splicing	rs1557522794	0	0	–	–	Disease causing	No	5.3	LP	Complex CMT2A,late-onset

*ExAC, Exome Aggregation Consortium; ACMG, American College of Medical Genetics and Genomics; CMT2A, Charcot–Marie–Tooth disease type 2A; LP, likely pathogenic; P, pathogenic*.

On the contrary, considerable phenotypic heterogeneity could be detected at the same position. The proband in F3 is a male patient diagnosed with slowly progressive dHMN with pyramidal signs carrying the p.Arg259Cys mutation while the male patient with the same mutation in F6 had a feature of motor dominant CMT2.

Furthermore, we also detected a case associated with p.GLn276His and p.Arg274Trp compound heterozygous variants. His mother was the proband of F10 with the p.GLn276His missense mutation. His father was an asymptomatic p.Arg274Trp variant carrier. Due to genetic burden effect, this patient experienced an earlier onset and more severe phenotype.

### Novel Variants Identified in our Cohort

**Figure 2:**
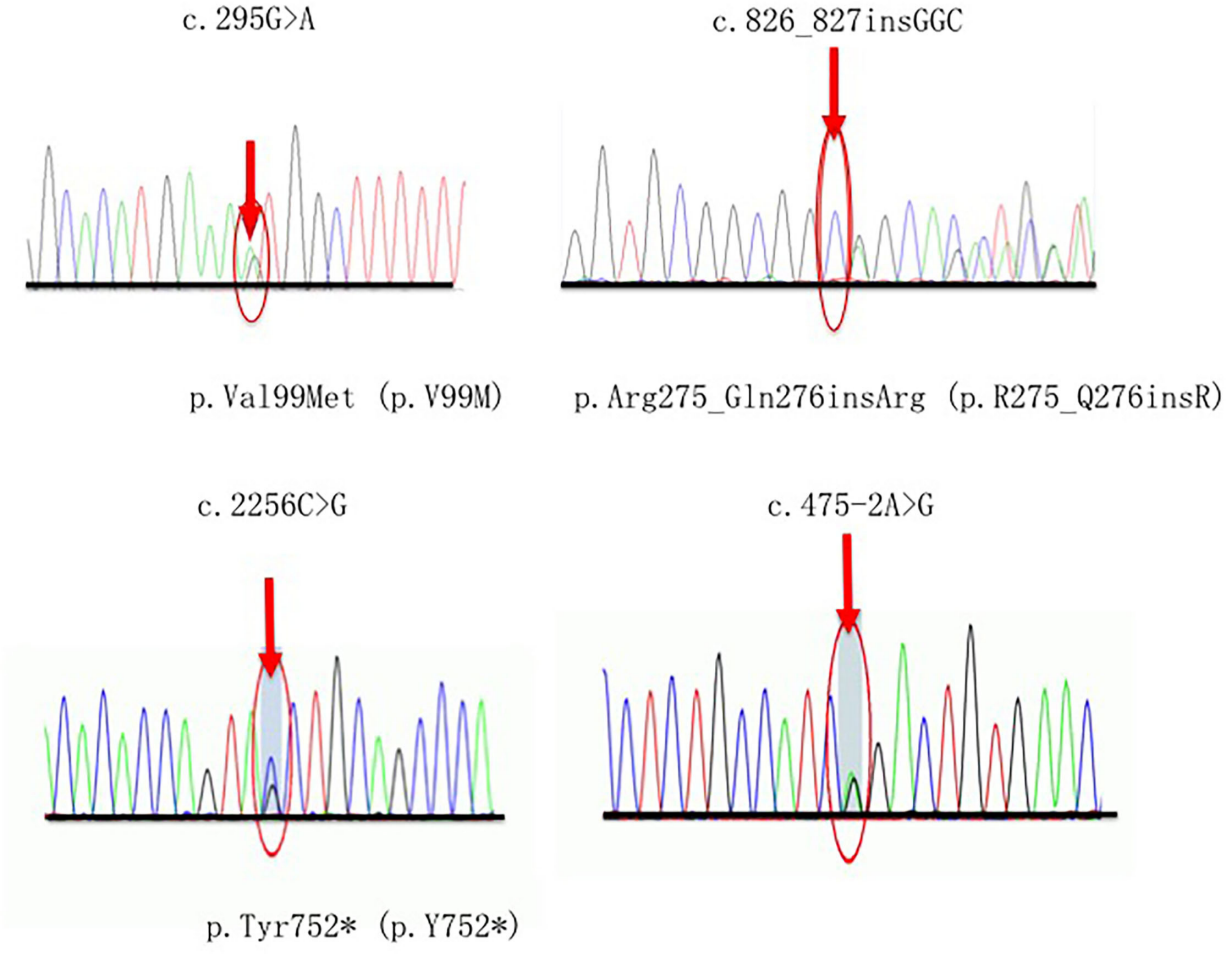
Sequencing chromatograms of four novel variants. Red arrows indicate the mutation site.

Four novel variants related CMT2A were detected in our study, in which one (p.Tyr752^*^) was classified as pathogenic (P), and the others (p.Val99Met, p.Arg275_Gln276insArg, c.475-2A>G) were considered as likely pathogenic (LP) according to the ACMG guidelines (14). Each of these variants occurs in an evolutionarily conserved region in the genes. Segregates were observed within two pedigrees (p.Val99Met, p.Arg275_Gln276insArg) (Figure 2).

A 19-year-old man who carried the *de novo* nonsense variant c.2256C>G (p.Tyr752^*^) presented with weakness in the lower limbs starting at 5 years old. Then the symptoms developed with weakness and atrophy of the upper extremity, foot deformity, occasional numbness, and hearing loss. He also had twice ankle surgery due to the severity of foot symptoms. His parents and older sister showed no evidence of the same MFN2 variants and neuropathy.

The heterozygous splicing variant c.475-2A>G in intro 5 was detected in a patient with late-onset and slowly progressive features. At the age of 54 years old, he presented with gait disturbance and weakness in foot flexion but sparing the upper limbs. He also reported pyramidal signs and, thus, classified as complex CMT2A.

A frameshift variant c.826_827insGGC (p.R275_Q276insR) was first detected in our cohort in a 16-year-old man with a feature of moderate sensorimotor peripheral neuropathy. His family presented a typical dominant inheritance pattern.

The proband in F7 was diagnosed as pure CMT2A with a novel missense variant p.Val99Met. She had a late-onset and mild phenotype, inherited from her mother who presented the similar symptoms at the age of 26.

### Characteristics of *de novo* Variants

We observed 35.0% (7/20) *de novo* variants (Arg94Trp, Lys307Glu, Gln367Pro, Ser249Phe, Pro251Ser, Val244Leu, Tyr752^*^) in our study. As *de novo* variants may have some special clinical characteristics, we did a comparison with the non-*de novo* group Table 4.

**Table 4:** The comparison between *de novo* group and non-*de novo* group.

	***De novo* (*n* = 7)**	**Non-*de novo*(*n* = 13)**	***p*-valve**
Age at onset (years)	4	13	0.021^*^
Gender (M:F)	5:2	7:6	0.642
CMTNS (median) (range)	12 (8–19)	11.5 (5–18)	0.692
CMTNS-G (median) (range)	1.36 (0.86–4)	1.24 (0.28–2.5)	0.371
Median CMAP (mean ± SE)	5.14 ± 1.33	6.90 ± 1.20	0.304
Median MCV (mean ± SE)	55.63 ± 4.42	59.16 ± 2.10	0.604
Ulnar CMAP (mean ± SE)	4.39 ± 2.72	4.37 ± 0.84	0.909
Ulnar MCV (mean ± SE)	49.80 ± 6.30	56.41 ± 1.88	0.400
Median SNAP (mean ± SE)	5.00 ± 0.50	4.22 ± 0.86	0.436
Median SCV (mean ± SE)	46.30 ± 5.20	49.98 ± 1.44	0.582

* *The values have significant difference (p < 0.05). CMTNS, Charcot–Marie–Tooth disease Neuropathy Scores; CMTNS-G, Charcot–Marie–Tooth disease neuropathy scores gradient; CMAP, compound muscle action potential; SNAP, sensory nerve action potential; MCV, motor nerve conduction velocity; SCV, sensory nerve conduction velocity*.

**Figure 3:**
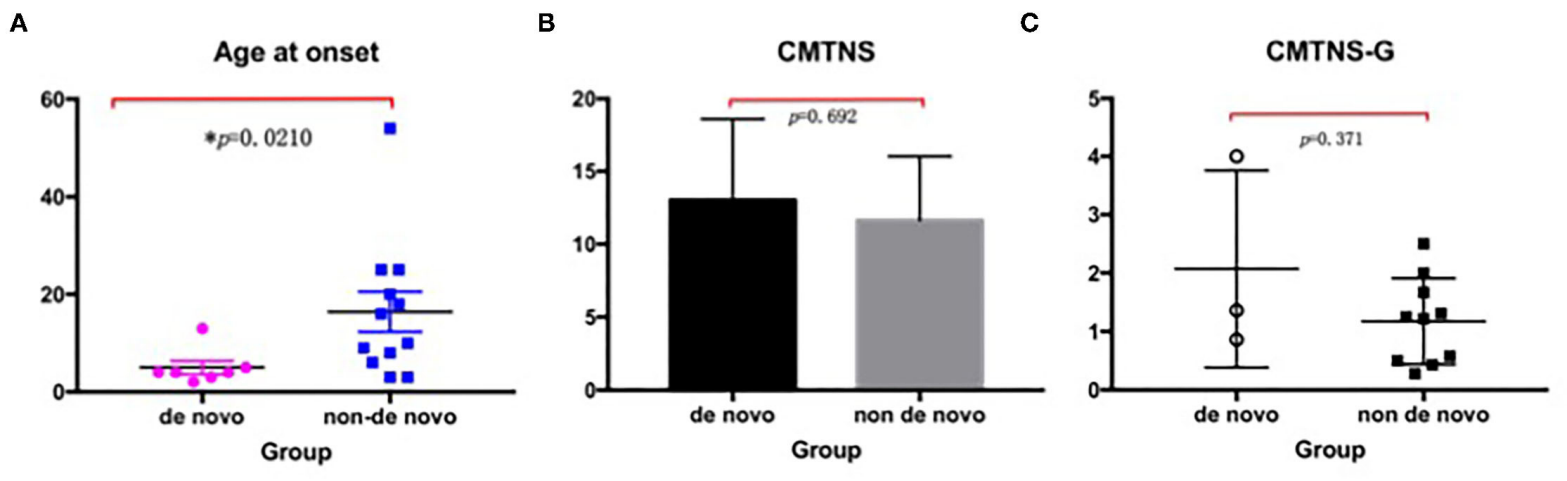
Comparison between the *de novo* group with the non-*de novo* group. **(A)** The median age at onset in the *de novo* group was significantly lower than that in the non-*de novo* group (*p* = 0.021). **(B)** The CMTNS showed no significant differences between the two groups (*p* = 0.692). **(C)** The CMTNS-G between the two groups showed no significant differences (*p* = 0371). CMTNS, Charcot–Marie–Tooth disease neuropathy scores; CMTNS-G, Charcot–Marie–Tooth disease neuropathy scores gradient.

The median AAO was 4 (2–13) years in the *de novo* group, which was significantly different from that of 13 (3–54) years in the non-*de novo* group (*p* = 0.021). The gender between the two groups showed no significant differences (*p* > 0.05). When the categories of disease severity were applied with CMTNS, the *de novo* group and the non-*de novo* group showed no significant differences (*p* = 0.692) (Figure 3).

The same situation can be seen when evaluated by CMTNS-G: the *de novo* group exhibited similar disease course compared with the *de novo* group (*p* = 0.371). Further comparison with neurophysiology showed that the *de novo* group presented a trend of severer nerve damage, with a lower CMAP and MCV in the median/ulnar nerve.

## Discussion

In this study, we identified 20 isolated families harboring 16 MFN2 variants in a large group of Chinese patients. The frequency of MFN2 variants was 5.0% (20/402), which was consistent with other reports (15). The extensive analysis of MFN2 variants and genotype–phenotype survey illustrated the features of Chinese MFN2 variants patients. MFN2 mutation caused the most common and typical CMT2 (the most popular form of axonal CMT). It has been reported that the frequency of MFN2 mutation ranged remarkably different in Spanish (16%) (5), French (18%) (6), Korean (23%) (7), Japan (8%) (2), Mainland China (18%) (1), Taiwan (14%) (3), and worldwide (30%) (4) CMT2 populations, due to different included criteria. In our cohort, the contribution of MFN2 variants to CMT2 is 12.6%, which is lower than that in previous southern Chinese report (1). The reason may be related to geographical and inclusion criteria bias.

The majority of patients (78.9%) developed symptoms in their childhood (<20 years old), which is the same as that worldwide (80.4%) (15). Based on the onset age, MFN2 variants tended to be earlier onset than other CMT subtype (9). Clinically, our patients were characterized by more involvement of the upper extremities (38.9 vs. 28.6%) and more sensory deficits (66.7 vs. 47.8%) than those in previous Chinese reports (1, 16). To simplify clinical features, we divided CMT2A into pure form (68.4%, 13/19) and complex form, depending on whether there is involvement other than axons of sensorimotor nerves. Pyramid sign was not common in our cohort, which can be detected in p.Arg94Trp, p.Pro251Ser, p.Tyr752^*^, c.475-2A>G, and p.Gln367Pro variants. When using CMTNS to reflect the severity, results are highly variable among cohorts. CMTNS in our study were below 20 (median = 12), regardless of the AAO. Therefore, our CMT2A patients seemed not severe, which was in accordance with a previous international study (CMTNS = 14.3) (4) but different from that of an American study (9). In comparison, another study in America revealed that MFN2 mutations caused severe phenotypes with a mean CMTNS of 21. This discrepancy among studies may be related to the preponderance of the early-onset and severe form in the American study and also the small CMTNS data we collected.

The electrophysiological study illustrated that our MFN2 variants were all axonal type. Reduced CMAP and SNAP amplitudes were predominant in the lower limbs. Moreover, we observed a tendency that median nerve was more severely affected than the ulnar nerve.

We identified 12 reported MFN2 mutations and four novel variants in our cohort. The most frequent variant was c.280C>T (p.Arg94Trp) in exon 4, which is highly conserved and located immediately upstream of the GTPase domain. In our study, most of the variants are located in or in close vicinity of the GTPase domains (amino acid positions 93–342), which is essential in the fusion process. Although variants in GTPase domain are in the majority, there is no difference in phenotypes compared with the other variants outside the GTPase domain.

The consistency of genotype and phenotype can be seen in some certain amino acid positions in MFN2 variants. For example, the missense variant p.Arg94Trp/Gln has a feature of early onset, high penetrance and tends to have a *de novo* origin, which are in line with previously published studies (15, 16). However, phenotypic heterogeneity is also evidence dependent on the amino acid substitution at the same position. According to previously published studies, the variant p.Arg364Trp has a feature of early onset and severe progression, while p.Arg364Gln illustrated a late onset phenotype (15). In addition, interfamilial phenotypic heterogeneity could also be detected in our study. The p.Arg259Cys variant in one family caused the phenotype of dHMN with pyramidal signs, while in the other family, the phenotype is motor predominant CMT2A.

The 31.6% of patients (p.Arg94Trp, p.Arg259Cys, p.Pro251Ser, p.Tyr752^*^, p.Gln367Pro, and c.475-2A>G) manifested a pyramidal sign with extensor plantar response or increased reflexes. MFN2-related CMT with pyramidal features are evidence in literatures (17). In general, pyramidal signs varied from extensor plantar response, preserved or increased reflexes to slightly increase in tone without frank spasticity. These atypical upper motor involvement signs may be related to the ubiquitous expression of *Mitofusin2 in vivo*, which play a fundamental role in the dynamic mitochondrial remodeling process governed by mitochondrial fusion and fission (18).

In addition, we also described a case associated with compound heterozygous variants (p.GLn276His p.Arg274Trp) in an autosomal dominant inherited family. Although both the missense variants were reported in literature (5, 19), the p.GLn276His variant might play a key role pathologically. On the contrary, the p.Arg274Trp variant might be a double-dose or genetic burden effect which made the phenotype more severe and earlier onset.

In our study, we detected four novel heterozygous variants. The nonsense variant c.2256C>G (p.Tyr752^*^), which is located in the HR2 domain near the C terminus caused a slightly truncated protein (losing six amino acids and <10% protein missing). Structural analysis suggested that this domain is essential for the fusion of the outer mitochondrial membranes and highly conserved (20). Till now, the same amino acid location mutation caused by a different nucleotide change c.2256C>A (p.Tyr752^*^) has been reported to cause a severe, early-onset axonal neuropathy. However, except for the severe CMT2 subtype, our patient also had hearing loss, indicating the genetic heterogeneity in MFN2 mutation. Functional analysis revealed that this mutation might cause a deletion of six amino acids and affect MFN2 expression.

The splicing alteration in intro 5 seems to be the hotspot splicing location in our study. The novel splicing variant c.475-2A>G exhibited late onset and moderate phenotype. Besides, we also detected the known mutation c.475-1G>T in another patient. The same as another Han Chinese cohort reported, our patient presented an adult-onset, mild progressive weakness of the distal lower limbs without a complaint of sensory abnormal. According to literature analysis, this splicing mutation may result a four amino acid residues deletion (p. T159_Q162del), which may explain his relatively mild polyneuropathy.

In our cohort, two groups of patients could be identified based on whether the variants were of *de novo* origins. Previous studies indicated that *de novo* variants were not rare in CMT2A families, especially in Asian populations. A Chinese group proved five out of 17 CMT2A pedigrees (29%) with an early onset and moderate phenotype (1). In other series, *de novo* variants account for 14.4% in Japan (21), 33% in Korea (10), 34% in America (22), and 63% in Britain (1), respectively. In this study, we confirmed the high frequency (35.0%) of *de novo* variants in CMT2A families, which is in accordance with previously published studies. *De novo* variants are a common disease mechanism in some childhood onset inherited diseases, which is associated with a reduced life expectancy and reduced reproductive fitness (23). Reproductive fitness is more common in early-onset severe genetic diseases, which is not conducive to genetic evolution and difficult to be passed on. The possible explanations for high frequency of *de novo* variants may include instability of mutation regions, environmental influence, or the ages of parents. Taken together, this high rate of *de novo* variants challenges the diagnosis of CMT2A as it is difficult to be distinguished from an idiopathic axonal neuropathy when there is no family history. Therefore, it may be useful to extend mutation screenings of MFN2 in this situation. Mutation p.Arg94Trp has been described as *de novo* in literature (1, 8) with an early onset phenotype, which is inconsistent with our findings. Besides, compared with patients in non-*de novo* group, patients with *de novo* variants experienced an earlier onset phenotype. Hence, *de novo* MFN2 mutations cause earlier onset phenotypes in CMT2A patients.

## Conclusion

In conclusion, this study represents a larger effort to study MFN2 variants to date in a Chinese population. Detailed analyses revealed that Chinese CMT2A is predominant typical pure CMT2A, with early onset and moderate phenotype. Of note that pyramidal signs were not uncommon in our cohort, it gave evidence for clinicians in directing future diagnosis and genetic screenings. Indeed, given the high frequency of *de novo* MFN2 variants, it is necessary to consider genetic study for patients with early onset and severe idiopathic axonal neuropathy.

## Data Availability Statement

The original data presented in the study are included in the article, further inquiries can be directed to the corresponding author.

## Ethics Statement

The studies involving human participants were reviewed and approved by the Institutional Ethics Committee of Peking University Third Hospital (PUTH). Written informed consent to participate in this study was provided by the participants or the participants legal guardian/next of kin. Written informed consent was obtained from the individual(s), and minor(s)' legal guardian/next of kin, for the publication of any potentially identifiable images or data included in this article.

## Author Contributions

DF and XL conceived and designed the study and reviewed and revised the manuscript. AS, YZ, and XL collected valuable clinical materials. YM and XL analyzed the data and wrote the manuscript. All authors read and approved the manuscript.

## Funding

This study was supported by the Key R&D plan of Department of Science and Technology (No. XZ202001ZY0005G) and Peking University Clinical + X youth program (2021–2022): PKU2021LCXQ019.

## Conflict of Interest

The authors declare that the research was conducted in the absence of any commercial or financial relationships that could be construed as a potential conflict of interest.

## Publisher's Note

All claims expressed in this article are solely those of the authors and do not necessarily represent those of their affiliated organizations, or those of the publisher, the editors and the reviewers. Any product that may be evaluated in this article, or claim that may be made by its manufacturer, is not guaranteed or endorsed by the publisher.
